# Impact of DRG reimbursement system on hospital efficiency: systematic review

**DOI:** 10.1186/1472-6963-12-S1-O2

**Published:** 2012-11-21

**Authors:** Hossein Moshiri Tabrizi

**Affiliations:** 1 United Nations University International Institute for Global Health (UNU-IIGH), Kuala Lumpur, Malaysia

## Background

During the last two decades, most countries including developed and developing have experienced a rapid increase in health care expenditures in general, and hospital expenditures in particular. DRG-based reimbursement systems were introduced to control healthcare and hospital expenditure, increase activity levels and standardize care. This paper reviews the theoretical and empirical evidence on whether DRGs can meet these ambitious objectives. The objective of this study is to systematically review the effect of DRG payment system on hospital efficiency and to find theoretical and empirical evidence that DRGs enhance efficiency and effectiveness in the hospital sector.

## Materials and methods

This study searched the EconLit and MedLine databases for published articles in the English between 1984 and 2009. Search terms included efficiency, Hospital efficiency, and frontier analysis. These could be reduced to more relate using additional keyword such as DRG reimbursement, DRG payment system. According to review objective relevant studies have been selected.

## Result

This paper reviewed 25 studies included all studies published in refereed journals or books that were either published or available in pre-print during the study period. According reviewed articles studies of the impact of DRGs on efficiency mostly focused on technical efficiency or productivity. The findings were somehow mixed, there was some evidence on improved technical efficiency (Portugal, Sweden, Norway) but nothing significant in the US, Austria as well. The country specific points and context may explain the divergent result.

## Conclusion

DRGs contributed to enhance understanding of the relationship between resource use and the activity in acute care setting. Evidence from empirical studies of the impact of DRGs reimbursement on hospital efficiency is mixed. While some research tentatively suggests efficiency improvements, at least in the short-run, attributing these to DRGs reimbursement is complicated by confounding factors.

